# Children Using Cochlear Implants Capitalize on Acoustical Hearing for Music Perception

**DOI:** 10.3389/fpsyg.2012.00425

**Published:** 2012-10-22

**Authors:** Talar Hopyan, Isabelle Peretz, Lisa P. Chan, Blake C. Papsin, Karen A. Gordon

**Affiliations:** ^1^Department of Otolaryngology, Cochlear Implant Program, The Hospital for Sick ChildrenToronto, ON, Canada; ^2^International Laboratory for Brain, Music and Sound Research, University of MontrealQuebec, ON, Canada; ^3^Department of Psychology, Ryerson UniversityToronto, ON, Canada

**Keywords:** auditory development, acoustical and electrical hearing, sensorineural deafness, hearing loss, cochlear implants, auditory plasticity, amusia, music perception

## Abstract

Cochlear implants (CIs) electrically stimulate the auditory nerve providing children who are deaf with access to speech and music. Because of device limitations, it was hypothesized that children using CIs develop abnormal perception of musical cues. Perception of pitch and rhythm as well as memory for music was measured by the children’s version of the Montreal Battery of Evaluation of Amusia (MBEA) in 23 unilateral CI users and 22 age-matched children with normal hearing. Children with CIs were less accurate than their normal hearing peers (*p* < 0.05). CI users were best able to discern rhythm changes (*p* < 0.01) and to remember musical pieces (*p* < 0.01). Contrary to expectations, abilities to hear cues in music improved as the age at implantation increased (*p* < 0.01). Because the children implanted at older ages also had better low frequency hearing prior to cochlear implantation and were able to use this hearing by wearing hearing aids. Access to early acoustical hearing in the lower frequency ranges appears to establish a base for music perception, which can be accessed with later electrical CI hearing.

## Introduction

Around the globe, music forms an integral part of society and culture, however, much of the acoustical information in music is unavailable to those with significant hearing loss or deafness without the aid of an auditory prosthesis. Cochlear implants (CIs) are neuroprosthetic devices that bypass the dysfunctional cochlea and directly stimulate the auditory nerve with electrical pulses. Children with congenital or acquired deafness who use CIs develop speech and language skills required for oral communication (Dawson et al., 1995; O’Donoghue et al., 1998; Spencer et al., 1998, 2003; Connor et al., 2000; Svirsky, 2000; Svirsky et al., 2000a,b,c; Szagun, 2000, 2001; Tomblin et al., 2000; El-Hakim et al., 2001a,b; Tyler et al., 2001; Geers, 2002; Geers et al., 2003b; Waltzman et al., 2003). Music perception is typically poor in individuals using CIs and the impact on music appreciation is often difficult to access particularly in children. In the present study, we used a tool designed to test music perception to identify which aspects of music can be heard by children using CIs.

### Cochlear implants distort musical cues

The perception of music relies heavily on detecting fine-grained pitch cues which are not well represented by CIs. Adult cochlear implant users have been consistently shown to have poor pitch perception in music, especially relative to performance on temporal-based tasks, such as detecting rhythm or meter differences (Gfeller and Lansing, 1991; Fujita and Ito, 1999; Cooper et al., 2008; Gfeller et al., 1997; Schulz and Kerber, 1994; Kong et al., 2004; McDermott, 2004; Looi et al., 2008). This is not surprising given that CIs cannot replace normal cochlear processing and do not provide a fully accurate representation of the original acoustic input, including fine temporal structure, intensity, and frequency cues of sound. In particular, the frequency composition is distorted because the implant parses sound into a limited number of frequency bands, does not encode the fine timing information (temporal fine structure) which the auditory system normally uses for frequency selectivity, and only delivers simulation in the basal portion of the cochlea (Rubinstein, 2004). Consequently, music may be difficult for cochlear implant users to listen to. Indeed, post-lingually deaf adult cochlear implant users typically describe music as unpleasant with a harsh sound quality and often dislike listening to music (Gfeller et al., 2000; Leal et al., 2003).

Studies investigating music perception in pediatric cochlear implant users suggest similar difficulties processing pitch information as reported by adult recipients, but less is known about the various components of music perception in children using CIs. Children using CIs can identify familiar music in its original form (i.e., with lyrics), but their listening is impaired when the same melody is presented in an instrumental version only (i.e., flute rendition; Nakata et al., 2005). The increased reliance on vocal information suggests that children using CIs have better access to speech sounds in music than to pitch and rhythm cues. Nonetheless, these children retain at least a basic perception of musical cues as shown by identification of happy and sad emotions in instrumental music (i.e., no lyrics) with accuracy well above chance (Hopyan et al., 2011). This distinction is likely made by using tempo cues (Hopyan et al., 2011). Despite hearing some musical cues, however, children using CIs continue to perform more poorly than typical hearing peers (Hopyan et al., 2011) and more poorly than adult CI users (Jung et al., 2012) on musical perception tasks. This indicates that these children do not have access to all of the acoustic information carried in music. What remains to be understood is what parts of music these children do and do not perceive. We hypothesized that, due to the impoverished frequency representation available through cochlear implant stimulation, pediatric cochlear implant users, like adult users, would have difficulty detecting differences in musical pitch relative to normal hearing peers. Because CIs process the temporal envelop of sound, we expected that pediatric cochlear implant users would have better access to rhythmic than pitch cues in music.

### Some children hear speech better with their cochlear implants than others

Cochlear implants provide access to hearing for all children who are deaf but some children are able to use these devices more effectively than others. We therefore asked whether it is possible to predict which children will be best able to perceive music through CIs.

This question has long been posed in the context of speech perception. Although no one factor explains the large variability in speech perception outcomes of cochlear implantation in children, the age at implantation has been shown repeatedly to be the most important predictor (Kirk et al., 2002; Sharma et al., 2005; Lesinski-Schiedat et al., 2006). Children implanted at ages less than 3 years show more rapid development of speech perception and better overall skills than their peers who are implanted at older ages (Hassanzadeh et al., 2002; Manrique et al., 2004; Tajudeen et al., 2010). Much of this has been ascribed to deafness-induced reorganization of the auditory thalamo-cortical pathways which occurs when the system is deprived of sound (Finney et al., 2001; Gordon et al., 2005; Sharma et al., 2005; Lee and Giraud, 2007). Such reorganization may be too extensive to reverse with cochlear implant stimulation. It is also possible that auditory plasticity decreases with increasing age. It has been difficult to distinguish whether increasing age, increasing duration of deafness, or both are responsible for poorer outcomes of cochlear implantation because many of the children studied were deaf from birth (i.e., their duration of deafness was equivalent to their age).

More recent changes to candidacy criteria for cochlear implantation might help to sort out the roles of deafness and age on developmental plasticity in the auditory system. Candidacy for cochlear implantation has changed over the years to include children who have some residual hearing. Early concerns were that implantation would destroy any remaining viable sensory cells in the cochlea but it became clear that, in many cases, this residual function was restricted to the low frequencies (i.e., 250–500 Hz) and was not supporting speech and language development. Most importantly, hearing aid use in some children yielded poorer speech perception skills than those achieved by many children using CIs (Somers, 1991; Geers and Moog, 1995; Meyer et al., 1998). As a result, CIs are now being provided to children who do have some residual hearing but these decisions are often delayed to ensure that any possibility of using hearing aids, which offer a non-surgical option and do not need to crudely covert sound into electrical pulses, is pursued. The delay to implantation in children who had some residual hearing means that CIs might have been provided at ages >7 years. In spite of several reports suggesting that cochlear implantation should not be done at these older ages, there is also evidence that some older children achieve good speech perception results (Dowell et al., 2002). We suspect that these good outcomes reflect auditory development promoted by hearing aid use in the period preceding cochlear implantation. That development would have, in effect, decreased the duration of time that the child was left deprived of sound. This too might have restricted the types of changes, which had been observed in individuals with more profound degrees of deafness. If so, this would mean that electrical hearing provided by CIs can take advantage of pathways primed with acoustic input delivered through acoustic amplification (i.e., hearing aids). On the other hand, it is not clear that the development of speech perception prior to cochlear implantation will help these children perceive music once they receive CIs. We therefore asked whether music perception for children using CIs would improve with better residual hearing in the unimplanted ear during the period prior to implantation.

## Materials and Methods

### Study participants

Twenty-three children and adolescents participated in the present study. They had used unilateral CIs for a mean(SD) duration of 6.4(3.4) years (CI group) and were between the ages of 7–16 years [mean(SD) = 12.5(2.7) years] and 22 age-matched typically developing control children with typical hearing [NH group; mean(SD) age at test = 11.8 (2.8) years].

The CI group consisted of 15 children and adolescents using right unilateral CIs, and eight children using left CIs. All CI children attended mainstream classrooms and used oral communication. Twenty-one CI participants used Nucleus 24 devices, 2 participants used Nucleus 22 devices, and 1 had an Advanced Bionics device. There were eight children in each group who had participated in musical training (35% of CI group and 36% of NH group). The duration of training was mean(SD) = 1.5(1.3) years for the eight children in the CI group and mean(SD) = 3.0(2.8) for the eight children in the NH group; these durations were not significantly different from one another [*t*(10) = 1.3, *p* = 0.21].

**Figure 1:**
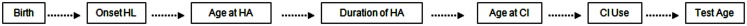
**Demographic factors occurring over a child’s life that could affect access to sound and auditory development**.

For children using CIs, the duration of deafness prior to cochlear implantation was calculated as the age of the child at implantation minus any time that the child had access to soft sounds and conversational speech. As shown in Figure 1, we identified demographic factors occurring over the children’s life time which would have affected their access to sound: the age at which bilateral severe to profound hearing loss was identified, when hearing aids were provided, whether hearing aids provided access to sounds ≤40 dB HL and for how long this remained the case prior to implantation. The degree of hearing and hearing loss in the CI group were ascertained through audiometric assessments in which behavioral measurements of hearing thresholds were determined in decibels relative to mean hearing thresholds (dB HL) for frequencies between 250 Hz and 4000 Hz both before and after cochlear implantation. Complete demographic data including thresholds achieved with hearing aids (aided thresholds) prior to implantation were unavailable in four children; however, in all cases, unaided thresholds before and after cochlear implantation confirmed profound bilateral hearing loss.

**Figure 2:**
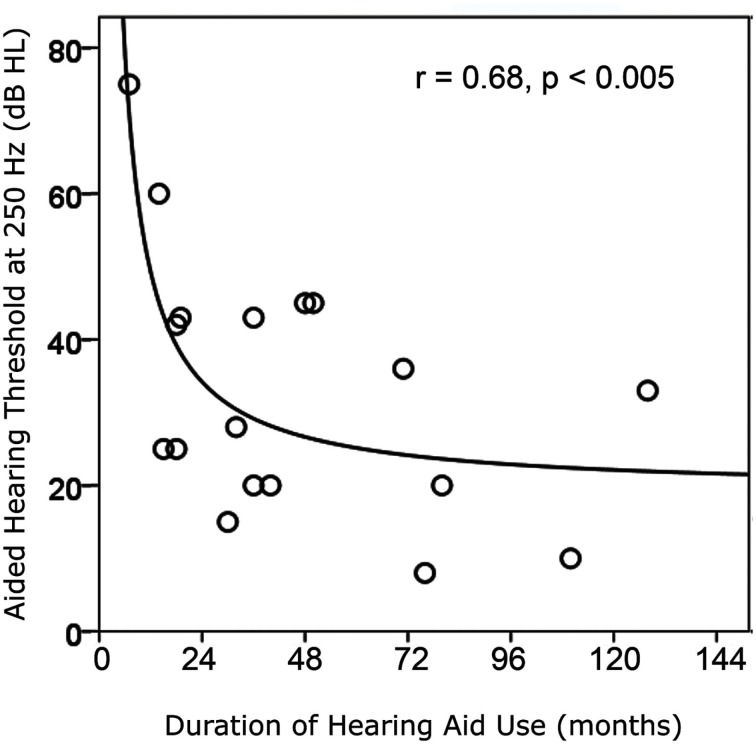
**Duration of hearing aid use prior to cochlear implantation increased as hearing at 250 Hz with the use of hearing aids improved (i.e., lower thresholds)**.

As expected, there was a negative association between pre-implant aided responses at 250 Hz and age at CI activation (*r* = −0.56, *p* > 0.05), showing that children who were older when they received their cochlear implant often had better residual hearing at this low frequency (decreasing thresholds in dB HL) prior to implantation. The children with better hearing at 250 Hz also tended to have progressive hearing loss as indicated by older ages when their hearing loss reached severe to profound degrees (*r* = −0.50, *p* = 0.031). The higher frequencies were most severely affected leaving the best hearing at 250 Hz [*F*(4, 12) = 10.1, *p* = 0.001]. We suggest that children with better residual hearing had greater access to sound prior to implantation and thus delayed seeking a cochlear implant. This is shown by a strongly positive correlation between duration of hearing aid use and the age at CI activation (*r* = 0.91, *p* = 0.0001) and, as shown in Figure 2, by improvements in aided hearing thresholds at 250 Hz with increasing duration of hearing aid use prior to implantation (*r* = 0.68, *p* = 0.002). These older children were also more likely to retain better residual hearing in the unimplanted ear after cochlear implantation (correlation between unaided hearing at 250 Hz and age at CI: *r* = −0.45, *p* = 0.03).

### Pretest assessment of task comprehension

The music tests in this study use a forced-choice task requiring participants to make distinctions between *same* and *different* melodies. As a result, a pretest consisting of a simple visual discrimination task was administered prior to the music discrimination tasks to ensure comprehension of instructions. Participants were presented with four geometrical shapes one at a time and asked whether they were the *same* or *different*. Following the successful completion of the pretest, the music tests were administered. Two practice trials were presented prior to each test with feedback provided, followed by 20 pairs of melodies. No feedback was given during the trials.

### Music tests

We used the child’s version of the Montreal Battery of Evaluation of Amusia (MBEA), which is a standardized measure of music perception originally developed for evaluating amusia in adults (Peretz et al., 2003). Amusia is a diagnostic label for individuals with normal hearing who are impaired in music processing. The MBEA is grounded in cognitive theories of music perception and neuropsychological evidence and has proven to be reliable, sensitive, and valid for assessing music processing in the adult population (Peretz et al., 2003).

The children’s version of the MBEA (Lebrun et al., 2012; Peretz et al., in review) consists of five tests each with 20 trials and 2 practice trials. The tests each measure a different component of music, all of which are necessary for normal perception of music. In each test, half of the trials consist of identical melodies, while the other half consist of different melodies. When different, a note has been either changed to an out-of-key note in the *scale* test, or to a note that changes the pitch directions in the *contour* test, or to a note that changes the intervals while maintaining the key and contour of the melody in the *interval* test. In the *rhythm* test, the grouping of note durations has been modified. For the Incidental *Memory* test, half of the trials contained melodies that were previously heard in the first four tests, while the other half contained new melodies. The melodies are computer-generated and each is delivered with the sound of a different instrument. Ten different sound timbres (e.g., piano, marimba, guitar, flute) are used to make the test as musically engaging as possible.

### Procedure apparatus and protocol

Musical stimuli were presented in a sound-proof booth and played through a laptop computer and an external speaker (UHL Studio Monitor 4406). Musical stimuli were in the range of 60–65 dB SPL as measured by a sound level meter placed in the approximate position of the participants head and played at a fixed volume for all participants. Participants were seated at a fixed distance, zero degrees azimuth to the speaker. No changes were made to their cochlear implant settings and none of the children wore a hearing aid in the opposite ear. Most of the children had severe to profound hearing loss in the unimplanted ear (*n* = 16) which meant that the music stimuli would be inaudible on that side. The other seven children had less severe hearing loss at 250 and 500 Hz (thresholds at 250 Hz = 45 ± 16 dB HL; thresholds at 500 Hz = 66 ± 19 dB HL).

Participants were asked to listen to brief pairs of music stimuli. Each trial commenced with a warning tone that specified the start of each trial, followed by a target melody, then a 2 s period of silence, and a comparison melody. Following each pair of music, participants are asked to indicate whether the two melodies were the *same* or *different*. For the final test, Incidental Memory, participants heard one piece of music at a time. After each trial, participants decided whether or not they had heard the piece of music in the preceding trials or if it was novel.

### Analyses

MBEA scores were analyzed in order to answer our two research questions. First, scores across subtests were compared using repeated measures analysis of variance (ANOVAs) with a between group analysis to compare data from the CI and NH groups. Second, effects of CI participant demographics on MBEA scores were assessed using linear regression models. Along with age at CI implantation [mean(SD) = 5.5(2.9) years], independent variables included in regression analyses were duration of deafness and degree of residual hearing. Effects of pre-implant hearing were confirmed by repeated measures ANOVA across subtest scores with a between group analysis of CI children with and without useable residual low frequency hearing (usable hearing at 250 Hz defined by thresholds of <40 dB HL with hearing aids).

## Results

### Music perception is abnormal in children using cochlear implants

**Figure 3:**
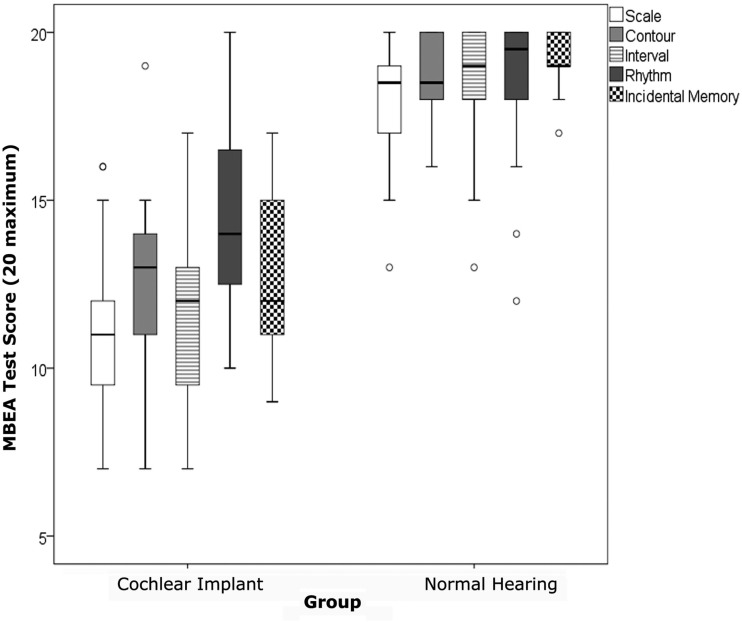
**Boxes indicate the data falling between the 25th and 75th percentile and the whiskers indicate the 95% confidence intervals**. The median scores are indicated by the thick black horizontal line. Scores on all tests were significantly better in the normal hearing group compared to the cochlear implant group. The cochlear implant group were most accurate on the Rhythm test.

We first examined whether children using CIs were able to detect differences in pitch and rhythm in music as well as their age-matched hearing peers. Group performance on MBEA tests are shown in Figure 3 (mean raw scores and standard deviation for CI group and NH groups). Figure 3 clearly shows differences between CI and NH groups with the NH group outperforming the CI group. Although the children with CIs performed above chance level (scores of 10 or above), they were clearly not performing at par with the NH group who were close to ceiling on all tests.

The CI group consisted of both left and right unilateral users. The determining factors for implanting the right ear vs. left ear are varied, including whether or not there is an intact cochlear nerve on one side vs. the other. In order to address the potential confound of the ear implanted, statistical analyses were conducted using a repeated measures ANOVA design to determine group differences within the CI group between left unilateral and right unilateral CI groups. No statistically significant differences were detected (*p* > 0.05) between the left and right unilateral CI groups. As a result, the two CI groups were combined. Repeated measures ANOVAs were conducted with MBEA tests (Scale, Contour, Interval, Rhythm, Incidental Memory) as *within-subjects* factors, group (CI and NH) as the *between-subjects* factor, and duration of musical training as a *co-variate*. Analyses revealed a significant main effect of MBEA tests, *F*(4, 10) = 7.4, *p* = 0.005, a significant effect of group, *F*(1, 13) = 31.5, *p* = < 0.0001, and a significant interaction effect of MBEA tests and group, *F*(4, 10) = 3.9, *p* = 0.04. There was no significant effect of music training *F*(1, 13) = 0.24, *p* = 0.63 nor significant interaction between music training and test, *F*(4, 10) = 3.9, *p* = 0.90. *Post hoc* analyses of simple effects for significant interaction showed that the CI group performed significantly less accurately across MBEA tests when compared to the NH group (*p* < 0.001). Within group analyses using repeated measures ANOVA with *post hoc* *t*-testing indicated that, as hypothesized, the CI group performed most accurately on the Rhythm Contour and least accurately on the Scale test [*F*(4, 19) = 3.8, *p* = 0.02]. The NH group also performed least accurately on the Scale test [*F*(4, 18) = 2.9, *p* = 0.049].

### Some children using cochlear implants perceive music better than others

**Figure 4:**
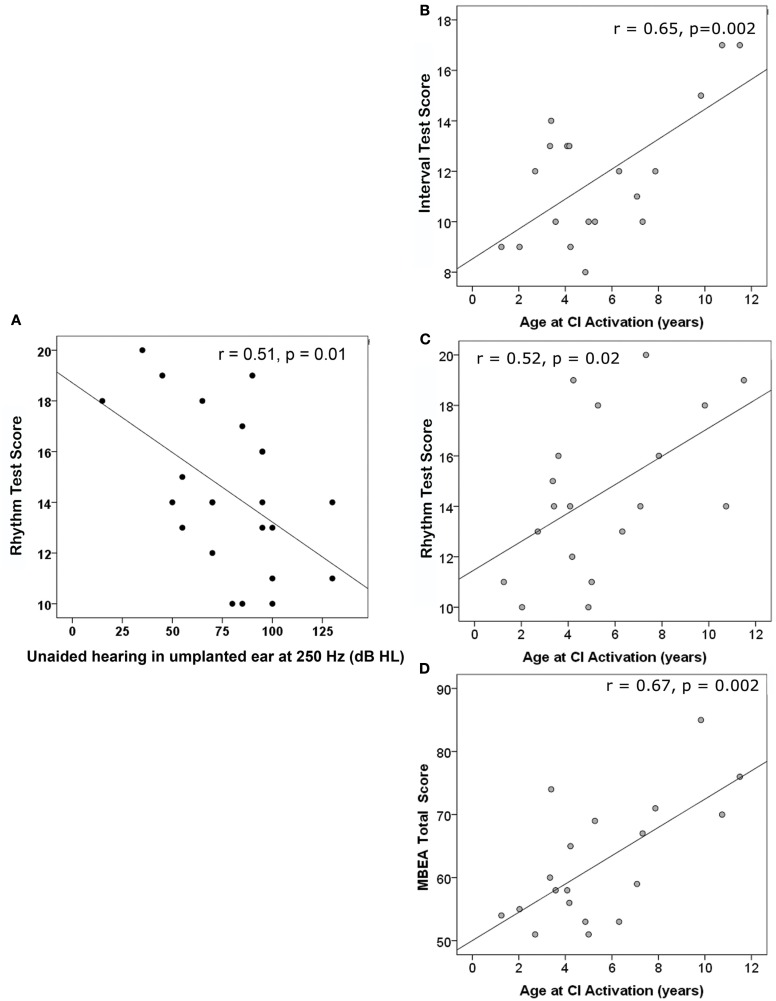
**(A)** Unaided residual hearing in the un-implanted ear during test was only correlated with one of the MBEA test scores; with better hearing at 250 Hz, Rhythm subtest scores improved. Three scores significantly improved as age at implantation increased: **(B)** Interval Test Score, **(C)** Rhythm Test Score, and **(D)** MBEA Total score.

The most obvious possible reason for improved scores in some children using CIs could be their use of residual hearing in the unimplanted ear. Yet, residual hearing on that side was only significantly correlated with scores on the Rhythm Test (*r* = −0.51, *p* = 0.01) and only for unaided thresholds at 250 Hz not 500, 1000, 2000, or 4000 Hz (*r* < |0.34|, *p* > 0.05). As shown in Figure 4A, scores on the Rhythm Test improved with decreases in unaided hearing thresholds (i.e., improved hearing) in the unimplanted ear at 250 Hz. Residual hearing in that ear (measured by unaided thresholds at 250, 500, 1000, 2000, and 4000 Hz) was limited and could not explain the variability in any of the other test scores (*r* < |0.30|, *p* > 0.05).

Because increasing age at implantation is known to result in poorer speech perception outcomes, we also assessed the relationship of test scores to age at CI activation. As shown in Figures 4B–D, there were significantly positive relationships between age at CI activation and scores on the Interval (4B) and Rhythm Tests (4C) and on the Total MBEA score (4D).

**Figure 5:**
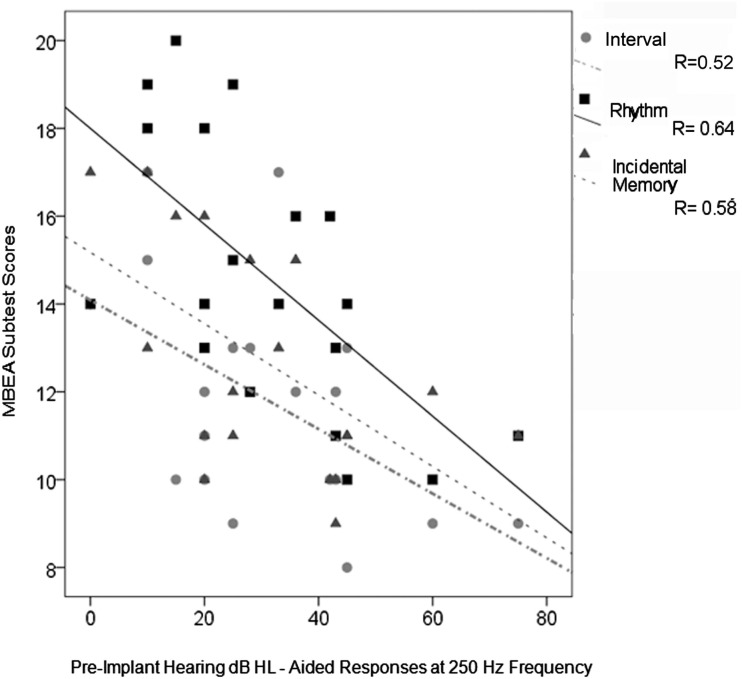
**MBEA total scores and test scores for Interval, Rhythm, and Incidental Memory significantly decreased with decreasing residual hearing (measured by higher aided thresholds at 250 Hz prior to cochlear implantation)**.

This was opposite to our expectations and could not be explained by longer periods of implant use in the older group since none of these scores were significantly related to duration of implant use (*r* > −0.40 and <0.19, *p* > 0.05) and, in multiple linear regression, age at CI activation continued to be significant (*p* < 0.05) whereas duration of CI use was not (*p* > 0.05). Rather, the positive effects of age reflected increased hearing aid use with better hearing thresholds prior to cochlear implantation; a repeated measures ANOVA indicated that children with aided thresholds at 250 Hz of better than 40 dB HL prior to implantation had significantly better subtest scores than children with poorer pre-implant hearing, *F*(1,17) = 5.9, *p* = 0.03. As shown in Figure 5, analyses of specific subtests revealed a significantly negative relationship between pre-implant aided residual hearing at 250 Hz and Interval (*r* = −0.52, *p* = 0.024), Rhythm (*r* = −0.64, *p* = 0.003), and Incidental Memory (*r* = −0.58, *p* = 0.009) tests. A significant relationship was also found on the MBEA total score (*r* = −0.67, *p* = 0.002).

In summary, some children using CIs were able to perceive musical cues better than others. Improved perception mainly in the area of musical rhythm and memory was found in children who had better residual hearing with longer periods of experience with acoustical hearing and, consequently, received their CIs at older ages.

## Discussion

### Abnormal music perception in children using cochlear implants

The current study evaluated music perception in deaf children using CIs. When compared to typically hearing age-matched control children, unilateral cochlear implant users performed significantly more poorly across all music tests of the children’s version of the MBEA task.

Children in the CI group had difficulty perceiving pitch-related information but showed relatively better performance on a temporal-based task (i.e., Rhythm test). Superior performance on rhythm relative to pitch perception is consistent with previous studies in adult CI users (e.g., Gfeller et al., 2000, 2002; Looi et al., 2004; Cooper et al., 2008). The inability to identify pitch information in music is mainly attributed to poor frequency resolution and lack of fine temporal structure provided by the cochlear implant device. This is not surprising given the limitations of cochlear implant technology in providing only gross spectral information with temporal envelopes processing up to a maximum of 22 frequency bands. However, despite relatively better performance within the CI group performance on the Rhythm test, they continued to perform significantly below that of the control group. Their poorer performance might be explained by the inclusion of pitch variation in the rhythm discrimination task. Indeed, when these pitch variations are removed, a music individuals who are impaired in melodic pitch processing, are able to normally discriminate rhythmic patterns (Foxton et al., 2006).

Another area of relative strength shown by the CI group was their performance on the Incidental Memory test on the MBEA, which was significantly better than other tests (i.e., eight children in the CI group scored 14 and above out of 20), although still significantly below the control group on this task. This indicates that these children had some ability to retain memory for melodies. This finding appears to be unique to children using CI as compared to adult CI users, who have been shown to perform poorly on the memory test of the adult version of the MBEA task (Cooper et al., 2008). Firstly, the Incidental Memory test on the MBEA as its name suggests, requires the retention and recognition of melodies presented over the duration of the test without advance warning of a memory component. As such, it captures the automatic retention as opposed to effortful retention of information. Secondly, memory for melodies involves the retention of both pitch and rhythm information. Based on previous and current findings that individuals with CIs are able to identify rhythm but not pitch, it is reasonable to assume that the CI group in this investigation relied more heavily on rhythm than pitch cues to recognize the melodies. In fact, the Rhythm and Memory test scores were significantly correlated in the CI group (e.g., *r* = 49, *p* = 0.03). Nonetheless, the CI group was able to automatically encode, retain, and retrieve complex auditory input without prompts.

It is unlikely that impaired performance on the MBEA task indicates an overall auditory memory deficit, since the task requires the retention of one melody in mind while listening and comparing it to the second melody. This line of thinking does not explain why the CI group scored significantly higher on the memory task. Children using CIs were able to retain melodic information and identify them at a later point in time despite being presented with a variety of timbres, albeit not as well as controls. Moreover, it does not explain why these children perform better on some subtests (i.e., Rhythm) than others.

### Residual hearing can support music perception with the cochlear implant

We found that outcomes on the MBEA Total and the Interval and Rhythm tests improve as the age of cochlear implantation increases. This appeared to counter evidence that children implanted at older ages generally achieve poorer speech perception skills than their peers implanted at younger ages (Hassanzadeh et al., 2002; Manrique et al., 2004; Tajudeen et al., 2010) and thus prompted further investigation into the hearing history of the CI cohort.

Importantly, as the age at CI increased, both the age at which severe to profound hearing loss occurred increased as well as the duration of hearing aid use prior to implantation. Some of these children might have waited a long time to obtain CIs because they had better low frequency hearing prior to CI. This finding reflects the expanding criteria of cochlear implant candidacy to include children who do have some residual hearing. At the same time, it shows the cautious approach that this cochlear implant center took in approving these children for cochlear implantation to ensure that they were no longer benefiting from their hearing aids prior to implantation.

Children implanted at older ages were more likely to retain some degree of low frequency hearing in the unimplanted ear as shown by a significant correlation between thresholds at 250 Hz and age at implantation. Although these children had limited access to the acoustic stimuli through their unimplanted ear, only the Rhythm scores were correlated with hearing thresholds (and only at 250 Hz). Rather, children with better access to acoustical hearing prior to cochlear implantation (aided thresholds at 250 Hz <40 dB HL) had significantly improved scores compared to children with poorer pre-implant hearing. This suggests that having greater access to acoustical hearing in the lower frequency range (i.e., 250 Hz) during early development enhances the perception of music with later cochlear implant use. Effects were found on the MBEA in general and, in particular, to tests of Interval, Rhythm, and Incidental Memory. Effects of pre-implant hearing on the Interval test, in particular, might be explained by development of auditory pathways through acoustical stimulation which can then be accessed with electrical stimulation. These findings are consistent with improved music perception in adults with post-lingual onset of deafness compared to children who have been deaf from young ages (Jung et al., 2012). This means that while cortical processing of spectral and temporal cues necessary for perceiving fundamental elements of music such as pitch are compromised by the cochlear implant (Limb et al., 2010), they are further restricted if the pathways never had access to acoustic hearing.

Access to acoustic input prior to implantation also appears to help children using CIs to remember melodic information. Memory for music is essential for deriving pleasure from music, and individuals often like familiar songs over novel songs. Children using CIs are known to take pleasure from music, are involved in musical activities (Gfeller et al., 1999; Stordahl, 2002; Nakata et al., 2005), and can recognize familiar songs (Vongpaisal et al., 2006; Trehub et al., 2009). By contrast, post-lingually adult CI users often qualify music as harsh sounding and dislike listening to music (Gfeller et al., 2000; Leal et al., 2003) and perform poorly on the musical memory task on the adult version of the MBEA task (Cooper et al., 2008).

## Conclusion

A remarkable demonstration of neural plasticity is the acquisition of sound perception in an otherwise silent brain in deaf individuals fitted with cochlear implant devices. The present findings suggest that exposure to early acoustical auditory experience enhances the perception of music with electrical hearing, especially for detecting differences in rhythm, and memorizing melodies. Even though children using CIs typically performed significantly poorer than their hearing peers in detecting musical cues some were able to perceive these cues better than others. Those children who had more access to acoustical hearing in the low frequency regions through their pre-implant hearing aids, used their hearing aids for a longer duration of time, received their implants at older ages, showed the best skills. This speaks to the importance of acoustic input for auditory development and suggests that these developing pathways can adapt to the new electrical input provided by a cochlear implant.

## Conflict of Interest Statement

The authors declare that the research was conducted in the absence of any commercial or financial relationships that could be construed as a potential conflict of interest.
